# Does Ether Assist Digestion?

**Published:** 1892-07

**Authors:** 


DOES ETHER ASSIST DIGESTION?—The effect of ether on digestive processes in healthy subjects, has been recently investigated by Dr. Gurieff, who gave thirty drops of sulphuric ether to six healthy persons during dinner, which consisted of about half a pint of soup, four ounces of meat, and six ounces of bread. It was found that the ether had the effect of stimulating the action of the gastric glands, increasing the free hydrochloric acid in the gastric juice and causing the peristaltic movements of the stomach, together with its power of absorption to increase thus; on the whole, exercising a favorable effect upon the gastric digestion. The same result was obtained when the ether was administered by means of hypodermic injections. It would appear, therefore, that the effects must be ascribed to a general, rather than to any merely local action on the mucous membrane of the stomach. Dr. Gurieff is disposed to think that there is a stimulation of the cephalic centers. This view is partly based on the observations of other Russian observers—Bekhtereff and Miloslevski and Pavloff and Shumova-Simanovskaya—on the dependence of the gastric functions upon the central nervous system.—*Lancet.*

